# Fluorescence Correlation Spectroscopy as a Versatile Method to Define Aptamer–Protein Interactions with Single-Molecule Sensitivity

**DOI:** 10.1021/acs.analchem.3c03341

**Published:** 2023-12-21

**Authors:** David Porciani, Manuela Maria Alampi, Stefania Abbruzzetti, Cristiano Viappiani, Pietro Delcanale

**Affiliations:** † MU Bond Life Sciences Center, University of Missouri-Columbia, 1201 Rollins Street, Columbia, Missouri 65211-7310, United States; ‡ Department of Molecular Microbiology & Immunology, School of Medicine, University of Missouri-Columbia, 1 Hospital Dr, Columbia, Missouri 65212, United States; § Dipartimento di Scienze Matematiche, Fisiche e Informatiche, Università di Parma, Parco Area delle Scienze 7A, Parma 43124, Italy

## Abstract

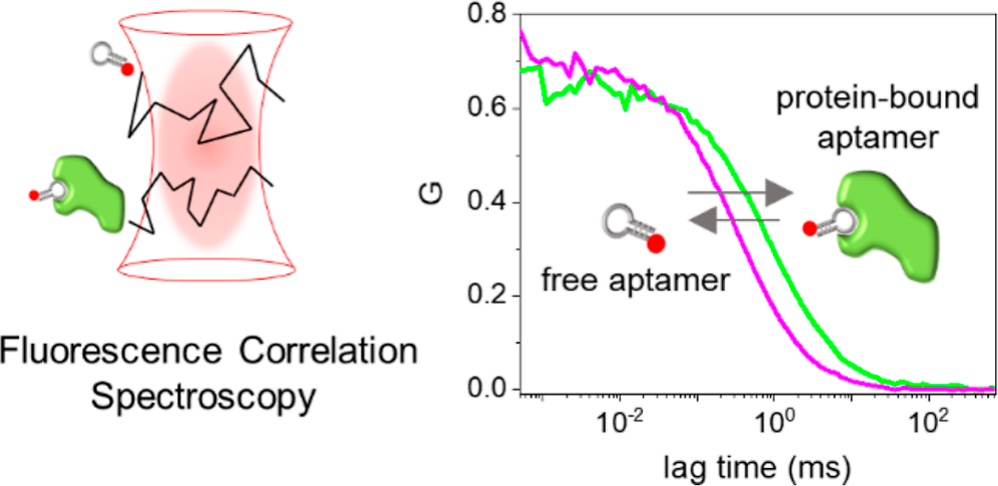

Aptamers are folded oligonucleotides that selectively recognize and bind a target and are consequently regarded as an emerging alternative to antibodies for sensing and therapeutic applications. The rational development of functional aptamers is strictly related to the accurate definition of molecular binding properties. Nevertheless, most of the methodologies employed to define binding affinities use bulk measurements. Here, we describe the use of fluorescence correlation spectroscopy (FCS) as a method with single-molecule sensitivity that quantitatively defines aptamer–protein binding. First, FCS was used to measure the equilibrium affinity between the CLN3 aptamer, conjugated with a dye, and its target, the c-Met protein. Equilibrium affinity was also determined for other functional aptamers targeting nucleolin and platelet-derived growth factors. Then, association and dissociation rates of CLN3 to/from the target protein were measured using FCS by monitoring the equilibration kinetics of the binding reaction in solution. Finally, FCS was exploited to investigate the behavior of CLN3 exposed to physiological concentrations of the most abundant serum proteins. Under these conditions, the aptamer showed negligible interactions with nontarget serum proteins while preserving its affinity for the c-Met. The presented results introduce FCS as an alternative or complementary analytical tool in aptamer research, particularly well-suited for the characterization of protein-targeting aptamers.

Aptamers are single-stranded RNA or DNA oligonucleotides that fold into a three-dimensional structure able to specifically bind a target molecule. Target-specific sequences are selected from an initial randomized library through a process named systematic evolution of ligands by exponential enrichment (SELEX).^1,2^ Aptamers have been selected against a large variety of targets, and they are considered a viable alternative to antibodies in sensing and therapeutic applications.^3–5^

The measurement of binding properties in vitro is the basis for the identification and optimization of functional aptamers. Equilibrium affinity for the target, expressed by the equilibrium dissociation constant (*K*_D_), is usually the main parameter evaluated to define the binding strength and to rank candidate sequences.^6^ Closely related to affinity, association and dissociation rates (*k*_on_ and *k*_off_, respectively) provide additional kinetic information, relevant to the engineering of aptamers with specific functionalities.^7,8^

Despite the availability of many analytical and biophysical techniques that quantify the binding affinity of aptamers,^9,10^ recent reports have highlighted the issue of poor or inconsistent characterization, suggesting the need for more stringent validation procedures.^11,12^ Additionally, besides the affinity for the target, evaluating the interactions with abundant non-target biological components, like serum proteins, is essential for applications.^6^ Because serum proteins have a high biological concentration (μM to mM), their impact on aptamer specificity,^13,14^ or pharmacokinetics^15–17^ is very relevant, even if affinities are typically weak.

Fluorescence correlation spectroscopy (FCS) provides quantitative binding characterization for various molecular systems.^18–20^ FCS measures the time that single fluorescent molecules employ to cross the detection volume of a confocal microscope when they move by three-dimensional diffusion within a solution. Binding is readily detected by monitoring changes in the diffusion time that occur when the fluorescent molecules interact with bulkier targets. Average diffusion times are not obtained from a bulk measurement but rather extracted from fluorescence fluctuations generated by the movement of individual molecules across the detection volume, providing high sensitivity and requiring small amounts of product. Additionally, unlike other biophysical techniques, FCS does not require the surface immobilization of molecules or their physical separation, e.g., by filtration or centrifugation. Notably, binding detection in biological fluids, like blood plasma, has been reported using specific setups.^21,22^ In the context of aptamer research, FCS appears particularly well-suited to study the interaction of fluorescently labeled aptamers with bulky proteins due to the large change in diffusion speed occurring upon complex formation.

Despite FCS being a viable tool for binding characterization and being able to circumvent specific limitations of other techniques, e.g., those associated with surface dynamics or the use of large sample volumes, it is essentially unused in aptamer research,^9,10^ with rare exceptions. FCS was applied to study the binding of an RNA aptamer to a dye-labeled moenomycin antibiotic.^23,24^ However, this approach required the conjugation of the small-molecule target with a dye, which introduces a major limitation. Following a different method, Zhou et al. developed a sensing assay based on a dual-color FCS setup to selectively detect thrombin by combining two dye-labeled aptamers targeting different epitopes.^25^ Still, this approach does not provide binding characterization, and it is hardly extended to other protein targets.

In this work, we propose a more general use of FCS as a tool for the characterization of aptamer–protein binding. We focused on the previously selected DNA aptamer, named CLN3, that targets the membrane protein receptor c-Met, also known as hepatocyte growth factor receptor.^26–28^ The CLN3 aptamer was chosen for our proof-of-principle study because its sequence has been tested and validated in previous reports, showing an affinity for the target in the nM range.^26–28^ Using FCS, we quantified the *K*_D_ of the binding between CLN3, labeled with a dye, and the soluble recombinant c-Met, and measured the corresponding binding kinetics. We then used FCS to evaluate the behavior of the aptamer exposed to physiological concentrations of the most abundant serum proteins. Finally, we measured the affinity between CLN3 and the protein target in a complex solution containing a mixture of serum proteins. The results introduce FCS as a tool for the binding characterization of aptamers against protein targets, both in pure solutions and complex environments.

## Materials and Methods

### Materials

Buffer components were obtained from Sigma-Aldrich. The final buffer used in FCS measurements is PBS pH = 7.4 enriched with 5 mM MgCl_2_ (or 1 mM for measurements on AS1411 and 36t aptamers) and 0.05% Tween-20. All buffers were sterilized in an autoclave. For FCS measurements, the sample was placed on glass coverslips #1 (Bio-optica) or LabTek 8-well chambers (Nunc).

DNA aptamer sequences (10–17 kDa) named CLN3 (5′-ATCAGGTGGATGGTAGCTCGGTCGGGGTGGGTGGGTTGGCAAGTCTGATTA-3′), G5mut (5′-ATCAGGCTGGATGGTAGCTCGGTCGATGTGGATGGTTTGTCAAGTCTGATTA-3′), AS1411 (5′-TTTGGTGGTGGTGGTTGTGGTGGTGGTGG-3′), and 36t (5′-CACAGGCTACGGCACGTAGAGCATCACCATGATCCTGTG-3′), directly conjugated with one atto633 dye at the 5′-end, were purchased from Integrated DNA Technologies (IDT). Oligonucleotide sequences were reconstituted in TE buffer (10 mM Tris HCl, 1 mM EDTA, pH = 8) to reach a stock concentration of 100 μM and stored at −20 °C. Concentrations were validated spectroscopically.

Recombinant human c-Met was purchased from R&D Systems (#358-MT/CF), reconstituted in PBS buffer pH = 7.4 to a stock concentration of 200 μg/mL, and stored at −20 °C. The molar concentration of the c-Met stock is 1.2 μM, calculated using a molecular weight of ∼170 kDa for the glycosylated protein (provided by the manufacturer). Molar concentration was validated spectroscopically using a molar extinction coefficient ε (280 nm) = 105,000 M^–1^ cm^–1^, calculated from the amino acid sequence. Recombinant human nucleolin (NCL) was purchased from Acrobiosystems (#NUL-H5253), reconstituted in water to a stock concentration of 400 μg/mL, and stored at −80 °C. The molar concentration of the stock is 2.8 μM, calculated using a molecular weight of ∼140 kDa for the glycosylated protein (provided by the manufacturer). Recombinant human platelet-derived growth factor B-chain homodimer (PDGF-BB) was purchased from Acrobiosystems (#PDB-H4112), reconstituted in 100 mM acetic acid to a stock concentration of 200 μg/mL, and stored at −80 °C. The molar concentration of the stock is 6.8 μM, calculated using a molecular weight of ∼30 kDa (provided by the manufacturer).

Human serum proteins were purchased from Sigma-Aldrich: fibrinogen (#F3879; ∼340 kDa), human serum albumin (HSA) (#A1653; ∼66 kDa), immunoglobulin-G (#I4506; ∼150 kDa), and transferrin (#T3309; ∼80 kDa). Highly concentrated serum protein solutions were prepared fresh before use by gently dissolving the protein in the final buffer. Molar concentrations were calculated from weight/volume and validated spectroscopically.

### Sample Preparation

Aptamer stock solutions were prediluted in TE buffer to 10 μM. Before the experiment, aptamers were further diluted to 100 nM in PBS buffer pH = 7.4 with 5 mM MgCl_2_ (or 1 mM for measurements on AS1411 and 36t aptamers), heated to 90 °C for 2 min in a thermomixer, and then cooled down to room temperature to promote correct folding. Folded aptamers were then diluted to a concentration of 1–2 nM in the final buffer.

For binding affinity measurements, varying concentrations of c-Met were titrated against a fixed concentration of CLN3 aptamer. An aliquot of c-Met stock solution 1.2 μM was thawed and serially diluted in PBS buffer to concentrations between 1000 and 1 nM. The same serial dilutions were prepared for the control with immunoglobulin-G. Then, final solutions (50 μL each) were prepared by mixing the folded aptamer (1 nM) with concentrated protein (between 1000 and 1 nM) in the final buffer to reach 100 pM of aptamer and 100–0.1 nM of protein. Solutions were incubated overnight at room temperature under dark conditions, with a gentle stirring. The same procedure was followed for titration experiments performed with NCL and PDGF-BB. PDGF-BB-containing solutions were supplemented with 1 mg/mL BSA.

For measurements performed exposing CLN3 aptamer to serum proteins, a highly concentrated solution of serum protein was prepared as described in Materials and serially diluted to the desired concentration range (μM to mM). Then, final solutions (100 μL each) were prepared by diluting the folded aptamer (100 nM) directly in the protein solutions (μM to mM) to reach a final aptamer concentration of 1 nM. Solutions were incubated overnight at room temperature under dark conditions, with a gentle stirring.

For binding affinity measurements performed in the presence of serum proteins, solutions of each serum protein at 5× the desired final concentrations were prepared as described in Materials. Then, equal volumes of the 5× concentrated solutions were mixed and centrifuged to remove aggregates (negligible in our measurements) to obtain a protein-mix solution. Serial dilutions of c-Met between 1000 and 1 nM were prepared in PBS buffer. Final solutions (50 μL each) were prepared by mixing the protein-mix solution with folded aptamer (2 nM) and c-Met (1–100 nM) in the final buffer. Final concentrations: aptamer 100 pM; c-Met between 100 and 0.1 nM; HSA ∼210 mg/mL; immunoglobulin-G ∼48 mg/mL; transferrin ∼12 mg/mL; fibrinogen ∼12 mg/mL. Solutions were incubated overnight at room temperature under dark conditions, with a gentle stirring.

For kinetic measurements, the CLN3–c-Met binding was monitored in time. The solutions (∼200 μL) were prepared directly in the well of an 8-well chamber by mixing folded aptamer (1 nM) and a 40–50× concentrated solution of c-Met in the final buffer to reach 100 pM of aptamer and 2 to 9 nM of protein. After careful mixing, the samples were immediately measured.

### FCS Instrumentation and Acquisition

FCS measurements were performed using a Microtime200 system from PicoQuant based on an inverted confocal microscope (Olympus IX70). Briefly, the emission from a 635 nm pulsed picosecond diode laser (maximum power on sample 24 μW, pulse frequency 20 MHz) is reflected by a dichroic mirror and focused on the sample by a 60× 1.2 NA water-immersion objective (UPlanSApo, Olympus). Fluorescence emission from atto633 is collected by the objective, crosses the dichroic mirror, and is selected with a band-pass filter (650–700 nm). After a pinhole (100 μm), the emission is split with a 50/50 splitter onto two single-photon avalanche diode (SPAD) detectors. In order to remove possible artifacts due to detector after-pulses, FCS autocorrelation curves were obtained by a cross-correlation between the time-resolved signal collected by the two SPADs. The acquisition time for a single measurement was set to 120 s for kinetics and 180 s for other measurements, with three repetitions. Binding titration experiments (as in Figure 2) were independently replicated four times and gave comparable results. The laser power on the sample was typically adjusted to ∼3 μW so that the photon count rate on each SPAD was ∼4000 counts per second, well above the dark level (200–400 counts per second), and kept constant. For kinetic measurements, FCS acquisitions were repeated on the same solution, which was placed in a well to avoid evaporation, at increasing times starting from the mixing of the solution. The total duration of the experiment was between 2 and 4 h.

**Figure 1:**
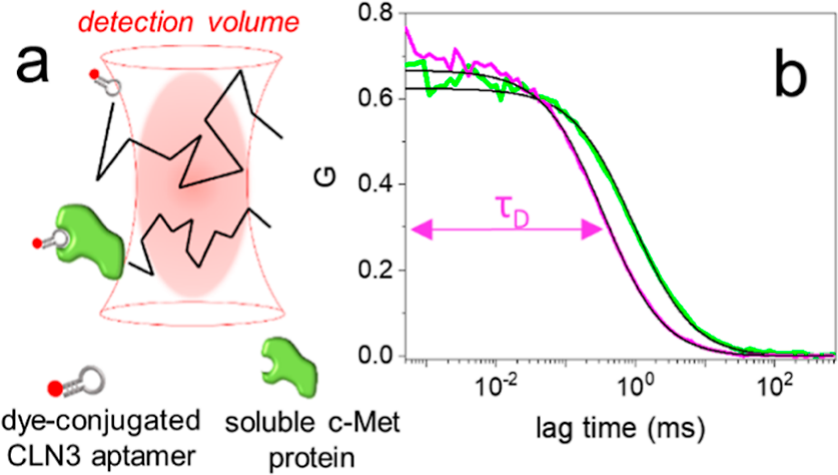
(a) Schematic representation of the principle of FCS binding measurements between the dye-conjugated aptamer and the target protein. (b) FCS autocorrelation curves measured for 1 nM CLN3-atto633, alone (magenta) and exposed to 200 nM of c-Met (green). The black lines represent the results of the fitting with a model comprising a single diffusing species (eq 1). The graphical representation of the diffusion time (τ_D_) obtained by the fit is highlighted for the magenta curve.

**Figure 2:**
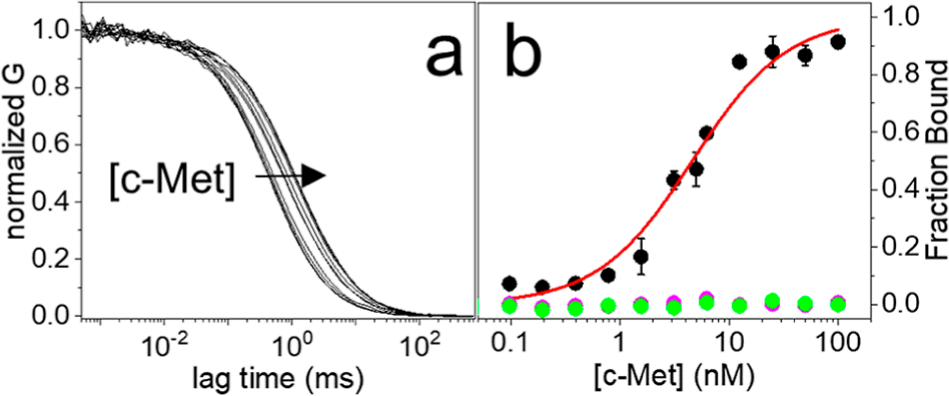
(a) FCS autocorrelation curves, normalized to 1, measured on solutions containing 100 pM of CLN3-atto633 and an increasing concentration of c-Met, from 0 to 100 nM. The change of the curves with c-Met concentration is highlighted by the arrow. (b) Corresponding data showing the calculated fraction of CLN3-atto633 bound to c-Met at increasing protein concentration (black). The red line shows the result of the fitting with a binding model (eq 5). The calculated fraction of protein-bound aptamer is shown for the control aptamer G5mut-atto633 exposed to c-Met (magenta) and for CLN3-atto633 exposed to IgG (green). Error bars: mean ± st. dev., 3 repetitions.

### Analysis of FCS Autocorrelation Curves

Autocorrelation curves were generated and analyzed with the software SymphoTime from PicoQuant. Autocorrelation curves *G*(τ) were fitted with a purely diffusive model comprising a single effective diffusing species
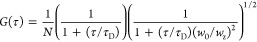
1where τ is the lag time, *N* is the average number of fluorescent diffusing species in the detection volume, τ_D_ is the diffusion time of the fluorescent species through the detection volume, and *w*_0_ and *w*_z_ are, respectively, the lateral and axial sizes of the instrument detection volume, yielding the structure parameter *k* = *w*_z_/*w*_0_. The effective detection volumes *V*_eff_ and *k* of our system were established with calibration and fixed during the fitting procedure: *V*_eff_ = 1.6 × 10^–15^ L; *k* = 6. The value of the three-dimensional diffusion coefficient (*D*) is directly calculated by the software from τ_D_ and the calibrated instrumental parameters, using τ_D_ = (*w*_0_)^2^/4*D*. Additionally, the instrument provides the average brightness of the detected diffusing species, calculated as the photon count rate divided by *N*.

Only for the autocorrelation curves in Figure 5, a purely diffusive model comprising two effective diffusing species was used for fitting
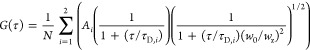
2where τ_D,*i*_ is the diffusion time of the *i*-th species and *A*_*i*_ represents the contribution of each species to *G*(τ). In this case, an average τ_D_ was calculated as the final output of the analysis
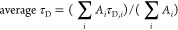
3

**Figure 3:**
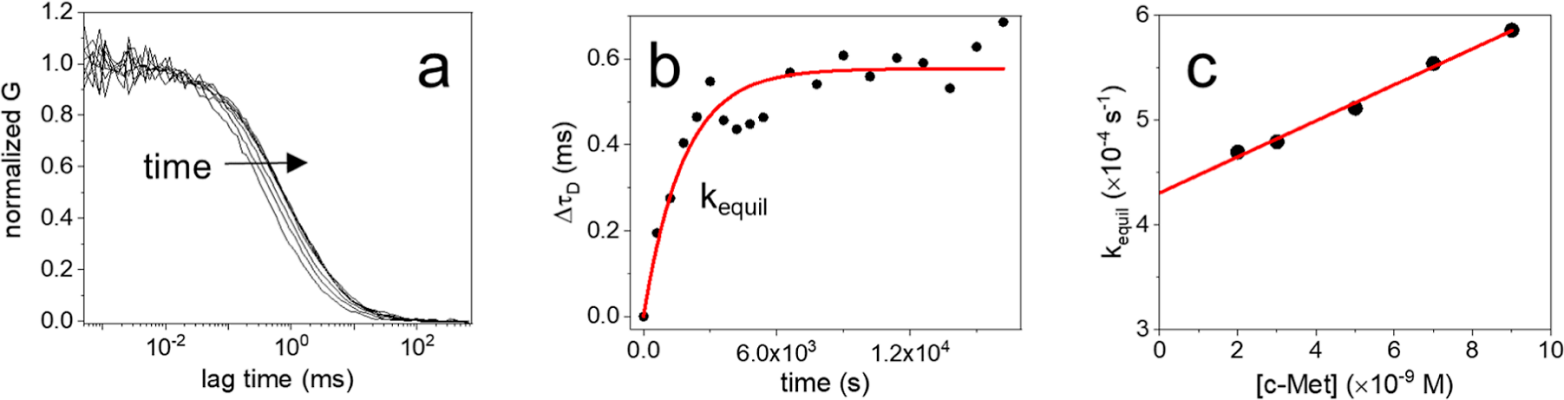
(a) FCS autocorrelation curves, normalized to 1, measured on a solution containing 100 pM of CLN3-atto633 and 5 nM of c-Met. The curves are measured at increasing times, from 0 to 120 min, starting from the initial mixing of the solution. The change of the curves in time is highlighted by the arrow. (b) Observed Δτ_D_ of the FCS autocorrelation curves measured at increasing times from the initial mixing of a solution containing 100 pM of CLN3-atto633 and 7 nM of c-Met (black). The red line shows the result of the fitting with an exponential model, yielding the *k*_equil_ parameter (eq 6). (c) *k*_equil_ values, obtained by the same procedure shown in panel (b), measured on solutions containing 100 pM of CLN3-atto633 and an increasing concentration of c-Met between 2 and 9 nM (black). The red line shows the result of the fitting with a linear model (eq 7).

**Figure 4:**
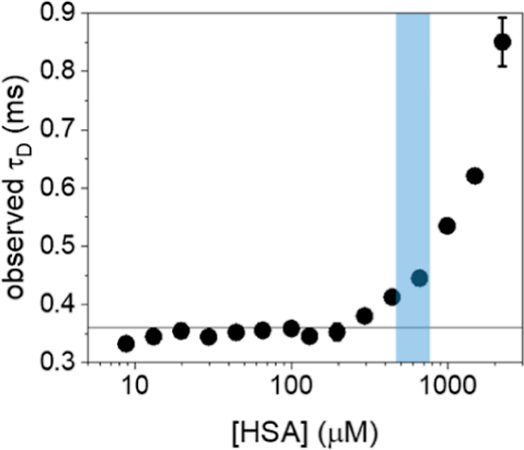
Observed τ_D_ values obtained on solutions containing 1 nM of CLN3-atto633 and an increasing concentration of HSA. The highlighted region indicates the range of HSA concentrations found in human serum.

**Figure 5:**
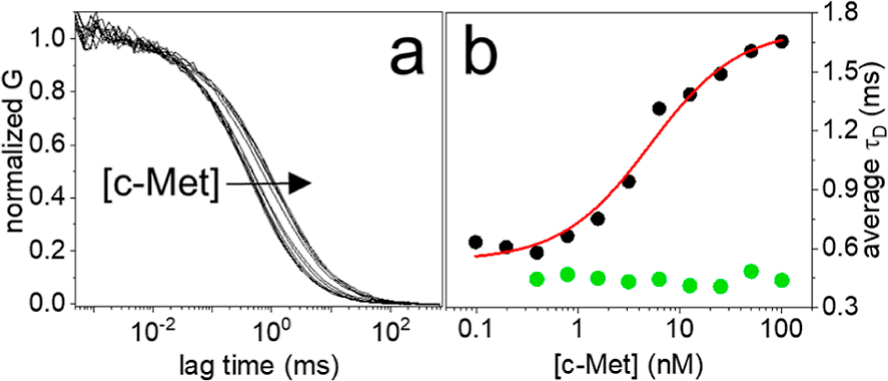
(a) FCS autocorrelation curves, normalized to 1, measured on heterogeneous solutions containing 100 pM of CLN3-atto633 and an increasing concentration of c-Met, from 0 to 100 nM, in the presence of HSA ∼210 mg/mL, IgG ∼48 mg/mL, Tf ∼12 mg/mL, and Fibr ∼12 mg/mL. The change of the curves with c-Met concentration is highlighted by the arrow. (b) Corresponding data showing the observed average τ_D_ at increasing c-Met concentration in the presence of serum proteins (black). The red line shows the result of the fitting with a binding model (eq 5). The results obtained from a control experiment with the G5mut-atto633 are shown (green). Error bars: mean ± st. dev., 3 repetitions.

### Analysis of Binding Curves and Kinetics

Binding curves for affinity measurements and kinetics were analyzed with the software Origin.

The fraction bound was calculated as
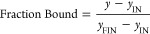
4where *y*_IN_ and *y*_FIN_ represent the initial and saturation values of τ_D_, respectively.

The binding curves of Figures 2 and 5 were fitted with the binding model
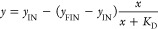
5where *x* is the concentration of protein, *K*_D_ is the equilibrium dissociation constant, *y* is either the fraction bound (Figure 2) or the measured τ_D_ (Figure 5), while *y*_IN_ and *y*_FIN_ either reduce to 0 and 1 (Figure 2) or represent the initial and saturation values of τ_D_, respectively (Figure 5).

The equilibration kinetics of Figures 3b and S7 were fitted with a monoexponential model
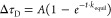
6where Δτ_D_ is the change of the diffusion time from the initial τ_D_ measured for the free aptamer, *A* is the maximum extent of this change, *t* is the time from the initial mixing of the solution, and *k*_equil_ is the equilibration rate.

The data of Figure 3c for the measurement of kinetic parameters were fitted with the linear model
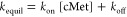
7where *k*_equil_ is the observed equilibration rate and *k*_on_ and *k*_off_ are the association and dissociation rates, respectively.

## Results and Discussion

### Detection of Aptamer–Protein Interaction with FCS

Figure 1a shows the principle of FCS measurement on aptamers. FCS measures the average time that a dye-conjugated CLN3 aptamer employs to cross the small detection volume of a confocal microscope (∼10^–15^ L) when it moves by three-dimensional Brownian diffusion within a homogeneous solution. If the aptamer binds the soluble c-Met protein target, a bulky fluorescent complex is formed, having a slower diffusion compared to the free aptamer. As a consequence, the measured time required by the protein-bound fluorescent aptamer to cross the detection volume shifts to longer values.

This effect can be easily appreciated in Figure 1b, which compares two autocorrelation curves, measured by FCS, obtained on solutions containing 1 nM atto633-conjugated CLN3 aptamer, either alone (magenta) or exposed to a 200-fold excess of the target c-Met protein (green). In the presence of c-Met, the autocorrelation curve is shifted toward longer lag times, consistent with the formation of bulky aptamer–protein complexes with slower diffusion. In order to quantify such changes, the diffusion time (τ_D_), representing the average time that fluorescent species employ to cross the detection volume, was found by fitting each autocorrelation curve with a model comprising a single effective diffusing species (eq 1, black lines). A τ_D_ = (0.37 ± 0.03) ms was found for the free aptamer, while a higher value, τ_D_ = (1.2 ± 0.1) ms, was found for the aptamer exposed to an excess of c-Met, consistent with the formation of slow-diffusing complexes. Absolute values of the three-dimensional diffusion coefficient (*D*) were then estimated from τ_D_ for the two solutions. A *D* ∼90 μm^2^/s was calculated for free CLN3, which is nicely consistent with *D* values reported for globular proteins of comparable molecular weight, such as myoglobin (*D* = 90 μm^2^/s^29^) or green fluorescent protein [*D* = (87 ± 2) μm^2^/s^30^]. A *D* ∼30 μm^2^/s was found for CLN3 exposed to an excess of c-Met, which was consistent with the *D* measured on a solution containing only c-Met, directly labeled with a fluorescent tag (Figure S1). These findings demonstrate that, under the employed conditions, nearly all the aptamers are bound to c-Met and that the complex diffusion is virtually identical to the diffusion of the c-Met protein, which is indeed much bulkier than the aptamer.

Figure 1b also shows that the two autocorrelation curves measured for free aptamer (magenta) and the aptamer–protein complex (green) have a comparable amplitude *G*(0). Because amplitude is related to the concentration of fluorescent species in solution (i.e., the free aptamer and the aptamer–protein complex), this observation indicates that CLN3 and c-Met form complexes with 1:1 stoichiometry under the employed conditions. This was also confirmed by an analysis of the brightness of the diffusing species, which gave very similar values for both the free aptamer and the complex (Figure S2). In fact, if more than one fluorescent aptamer were bound to a single c-Met, the complexes would have shown an increased brightness and a reduced apparent concentration in comparison to free aptamers.

The above findings demonstrate that FCS detects the interaction between CLN3 and c-Met with high sensitivity (see Figure S3 for a comparison with fluorescence anisotropy results), also providing absolute *D* values for free aptamer and complex.

Following the same procedure, FCS was also tested using two different DNA aptamers, namely AS1411^31,32^ and 36t,^33^ which respectively bind NCL and PDGF-BB. These protein targets have a completely different nature and very different sizes (NCL is ∼140 kDa; PDGF-BB is ∼30 kDa). In both cases, FCS clearly detected an increase in τ_D_ when the fluorescent aptamers were exposed to an excess of the protein target, reflecting the formation of stable complexes (Figure S4). These results indicate that the proposed FCS-based approach for binding detection can be successfully applied to various aptamer–protein pairs, including protein targets with moderate size and relatively fast diffusion, like PDGF-BB.

### Measurement of Binding Affinity

Having demonstrated that FCS readily detects the binding between CLN3 and the target c-Met protein, we performed an experiment to quantify the *K*_D_. To do that, 100 pM of fluorescent CLN3 were exposed to increasing concentrations of c-Met between 0 and 100 nM. Figure 2a shows the normalized autocorrelation curves, measured by FCS, where it is easily seen that the correlation shifts to longer lag times at increasing concentrations of c-Met, consistent with an increasing fraction of CLN3 that binds to the slow-diffusing protein. Even if the solutions contain two fluorescent species having different diffusion, i.e., the free aptamer and the complex, the autocorrelation curves were well-fitted with a model comprising a single effective diffusing species (eq 1), yielding an apparent τ_D_ value (representative results of the fit are shown in Figure S5). The fraction of protein-bound aptamer, or simply fraction bound, was then calculated by comparing the apparent τ_D_ obtained by the fit with reference values corresponding to the fully free aptamer and the fully protein-bound aptamer (eq 4).^34^Figure 2b shows in black the fraction of CLN3 bound to c-Met at increasing protein concentrations. The fraction bound grows significantly between 1 and 10 nM of c-Met. This trend was well-fitted with a simple binding model (eq 5, red line), yielding a *K*_D_ = 5 ± 1 nM. The obtained *K*_D_ is in line with the values measured during the selection and optimization of CLN3 by dot blot analysis of radiolabeled sequences^26^ and with a value obtained by biolayer interferometry.^35^

In order to verify that FCS correctly reports the specificity of the interaction between CLN3 and c-Met under the employed conditions, we performed two control experiments. In the first one, we used a mutated CLN3 aptamer, named G5mut, in which five key guanine residues involved in the formation of G-quadruplex structures^28^ were replaced with adenine or thymine. According to previous reports, the destabilization of G-quadruplexes in CLN3 suppresses the binding to c-Met, making the sequence essentially nonspecific.^27,28^ Exposing the dye-conjugated G5mut aptamer to c-Met at increasing concentrations up to 100 nM did not lead to any appreciable change in the measured τ_D_ and the calculated fraction bound (Figure 2b, magenta), proving that the mutated aptamer does not appreciably bind to the target. In a second experiment, we exposed the CLN3 aptamer to human immunoglobulin-G (IgG), a protein with molecular weight and diffusion properties (*D* ∼40 μm^2^/s^36,37^) comparable to those of the soluble c-Met. Also in this case, the measured τ_D_ and the calculated fraction bound values of CLN3 were not affected by the presence of IgG up to 100 nM (Figure 2b, green). These results confirm that the specificity of the CLN3 sequence for the employed c-Met protein is successfully reported by FCS.

Finally, the same FCS-based procedure was employed to measure the binding affinity of the aptamers AS1411 and 36t to NCL and PDGF-BB, respectively. A *K*_D_ = 30 nM was obtained for the AS1411-NCL pair (Figure S6a,b), consistent with previous measurements performed with surface plasmon resonance (*K*_D_ = 34.2 nM).^38^ The FCS measurements on the 36t aptamer and PDGF-BB highlighted a very high affinity, with sub-nM *K*_D_ (Figure S6c,d), roughly in line with previous characterizations (*K*_D_ ∼0.1 nM, by filter binding assays;^33^*K*_D_ ∼1 nM, by biolayer interferometry^39^). These findings indicate that the proposed approach can discriminate binding affinities within a wide range of *K*_D_ values (from ∼10–100 to ∼0.1 nM), even if the precise determination of sub-nM affinities is limited by the need to operate at pM concentrations of fluorescent aptamer, an experimental condition that leads to a reduced signal-to-noise ratio in FCS.

### Measurement of Binding Kinetics

The interesting point was, then, to understand whether FCS could be usefully exploited to measure binding kinetics (*k*_on_ and *k*_off_) between CLN3 and c-Met. Binding kinetics can be inferred from the equilibration rate (*k*_equil_), which is related to the time required to reach a stable binding equilibrium upon mixing.

When 100 pM of dye-conjugated CLN3 are mixed with 1–10 nM concentrations of c-Met, equilibration is relatively slow (tens of minutes to hours) compared to the acquisition time of an autocorrelation curve (2 min). This process was slow enough to be monitored using FCS by measuring autocorrelation curves on the same solution at different time points from the initial mixing. An example is shown in Figure 3a where it can be seen that the curves shift toward longer lag times with increasing time from the initial mixing until they reach stability, corresponding to a complete equilibration. The changes of the autocorrelation curves were then exploited to follow equilibration kinetics: the aptamer is initially unbound (i.e., fast diffusing), and the fraction of protein-bound aptamer (i.e., slow diffusing) grows in time, causing the observed shifting of the autocorrelation curves until a stable condition is reached.

In order to quantitatively describe this process, an apparent τ_D_ value was found by fitting each autocorrelation curve with a model comprising one effective diffusing species (eq 1). Then, the Δτ_D_ was calculated by comparing the apparent τ_D_ with the initial τ_D_ value of the free aptamer. Finally, Δτ_D_ is plotted in time, starting from the initial mixing of the solution, as shown in Figure 3b (black). Under the conditions employed, equilibration kinetics showed a fast formation of complexes, approximately in the first 60 min, corresponding to large changes in Δτ_D_, followed by a stabilization where Δτ_D_ fluctuates around a constant value (Figure 3b). The measured equilibration kinetics were then fitted with a single exponential model to obtain the *k*_equil_ value (eq 6, Figures 3b and S7). Importantly, a control measurement confirmed that experiments were not affected by photobleaching or nonspecific molecular adsorption on surfaces (Figure S8).

It is expected that the observed *k*_equil_ value linearly increases with the growing concentration of the target protein.^40^ This expected linear trend is nicely retrieved in our results, as displayed in Figure 3c (black). A fitting of these data with a simple linear model (eq 7 and Figure 3c) allows the discrimination of *k*_on_ and *k*_off_, corresponding to slope and intercept, respectively. A *k*_on_ = (1.70 × 10^4^) M^–1^ s^–1^ and a *k*_off_ = (4.30 × 10^–4^) s^–1^, corresponding to a complex half-life *t*_1/2_ = 27 min, are found for the binding between CLN3 and c-Met. The ratio *k*_off_/*k*_on_ yields a *K*_D_ ∼25 nM, higher than the value obtained from the data in Figure 2b. However, the *K*_D_ calculated from kinetics originates from a multistep data analysis, which is likely to increase the uncertainty compared to the more direct titration measurement of Figure 2. Remarkably, the *k*_off_ determined by FCS is consistent with a reported value measured with biolayer interferometry under comparable conditions [*k*_off_ = (5.43 × 10^–4^) s^–1^; *t*_1/2_ = 21 min].^35^ Conversely, biolayer interferometry gives a higher *k*_on_ (5.08 × 10^5^ M^–1^ s^–1^).^35^ This discrepancy is possibly due to the uncertainty related to the data analysis and/or to intrinsic differences in the measurement procedures of the techniques, including the surface-immobilization of molecules.

### Investigation of Aptamer Interactions with Serum Proteins

We then investigated whether FCS could be exploited to study the behavior of the CLN3 aptamer in the presence of four abundant human serum proteins with different biological functions: HSA, IgG, transferrin (Tf), and fibrinogen (Fibr). Because FCS operates on homogeneous solutions, protein concentration could be increased to physiologically relevant values (μM to mM), close to the conditions found in serum.

We first exposed 1 nM of dye-conjugated CLN3 aptamer to high concentrations of HSA, from 10 μM to 2 mM. The emission of the fluorescent aptamer was clearly detected above the background (Figure S9a), enabling the measurement of autocorrelation curves that were well-fitted with a model comprising a single effective diffusing species (eq 1). Figure 4 reports the τ_D_ values obtained at increasing HSA concentration. Up to ∼300 μM of HSA, τ_D_ remains consistent with the value of free aptamer, indicating no detectable binding. This demonstrates that the affinity between CLN3 and HSA is very low, with a *K*_D_ >300 μM. Above ∼300 μM of HSA, τ_D_ values increase significantly, reflecting a slowdown of aptamer diffusion. However, τ_D_ does not show saturation, as expected for bimolecular binding processes, but displays continuous growth with increasing HSA concentrations. This behavior is attributed to an increased viscosity of the solution, which causes an indefinite growth of the observed τ_D_ values without saturation. This hypothesis is in line with previous data reporting the viscosity changes of concentrated protein solutions.^41,42^ Additionally, a control experiment performed with dye-labeled albumin showed a trend of the observed τ_D_ that is nearly identical to that of CLN3 (Figure S10). This evidence suggests that the increase of τ_D_ observed at physiological concentrations of HSA (highlighted region in Figure 4) is mainly due to a viscosity change of the solution rather than to the formation of stable complexes. Still, the occurrence of a residual binding to HSA, with very low affinity, cannot be fully ruled out by these measurements.

Figure S11a shows the τ_D_ values measured for the dye-conjugated CLN3 exposed to IgG, between 1 and 100 μM. As observed for HSA, τ_D_ values tend to grow with increasing IgG without reaching saturation. Additionally, the τ_D_ values measured at physiological concentrations of IgG (τ_D_ ∼0.5 ms) are much lower than those expected for an antibody complex (∼1 ms) and remain closer to the value of the free aptamer. This evidence demonstrates that CLN3 is mostly unbound when exposed to physiological concentrations of IgG. The modest increase of the observed τ_D_ of the aptamer is possibly related to viscosity changes, as previously discussed for HSA, even if the existence of a weak interaction with IgG cannot be excluded.

Figure S11b,c shows the observed τ_D_ measured for the dye-conjugated CLN3 exposed to Tf, between 1 and 100 μM, and Fibr, between 1 and 10 μM. In both cases, the τ_D_ values remain approximately constant with increasing protein concentration, fluctuating around the reference value of the free aptamer. In particular, no detectable interaction is occurring between CLN3 and Tf or Fibr at physiological concentrations of these proteins.

Altogether, the above FCS results indicate that the CLN3 aptamer exposed to physiological concentrations of these serum proteins remains mostly unbound, even if weak interactions cannot be fully excluded.

### Affinity for the Target in the Presence of Serum Proteins

The previous results suggested that CLN3 has negligible binding when exposed to a single serum protein at physiological concentrations. However, biological fluids, such as serum, are heterogeneous and contain a variety of proteins that might interfere with target recognition.

In order to simulate this situation, we used FCS to investigate if the aptamer retains its ability to bind the c-Met target within a heterogeneous solution of serum proteins. To do so, we first prepared a solution containing HSA, IgG, Tf, and Fibr at concentrations within the range found in human serum. In spite of the complexity of the solution, by introducing 100 pM of dye-conjugated CLN3 aptamer in the protein solution, the fluorescence signal could be clearly detected above the background (Figure S9b), enabling quantitative measurements. A further control experiment confirmed that the signal from the fluorescent aptamer could be discriminated also within other types of complex solutions (Figure S12). We then performed a titration in which 100 pM of CLN3 were exposed to increasing concentrations of the target protein c-Met, between 0.1 and 100 nM, while HSA, IgG, Tf, and Fibr were kept at the same physiologically relevant concentrations. As shown in Figure 5a, the autocorrelation curves measured by FCS clearly shifted toward longer lag times with increasing concentrations of the c-Met protein. This indicates that the concentration of c-Met correlates with a growing fraction of species that have slower diffusion compared to the solution without c-Met. Because the concentration of serum proteins remains constant in all the measurements, the change in the autocorrelation curve reflects the formation of stable slow-diffusing complexes between CLN3 and c-Met, as previously observed in pure solutions (Figure 2a).

The curves in Figure 5a were well-fitted with a model comprising two effective diffusing species (eq 2). Due to the heterogeneity of the solutions, it was hard to consistently assign these two species to a unique molecular counterpart with a well-defined diffusive behavior. We therefore considered a single effective τ_D_ value obtained by averaging the contributions of the two species comprised in the fitting model (eq 3). Figure 5b shows the values of the average effective τ_D_ obtained for increasing concentrations of c-Met. While it is difficult to quantitatively relate such average τ_D_ values to the fraction of target-bound aptamer, these values are a useful tool to describe the changes reported by FCS autocorrelation curves. Qualitatively, the average τ_D_ values of Figure 5b show the trend of growth typical of bimolecular binding, with a significant increase between 1 and 10 nM of c-Met, followed by a saturation at higher protein concentrations. In line with this observation, the data were well-fitted using a binding model (eq 5, Figure 5b) that yielded a *K*_D_ = 5 nM. Notably, this value is perfectly consistent with the *K*_D_ measured for CLN3 and c-Met in a pure solution (Figure 2). The fact that the measured equilibrium affinity for the c-Met target remains the same in the presence of a mixture of serum proteins at physiologically relevant concentrations indicates a negligible effect of these serum components on target recognition, at least under the employed conditions. This fact is also consistent with a negligible or very low affinity between CLN3 and the main serum proteins, as reported in Figures 4 and S11.

Finally, the data shown in green in Figure 5b display the results of a control experiment obtained using the nonspecific aptamer G5mut instead of CLN3, while keeping identical experimental conditions and the same analysis procedure. The observed average τ_D_ values do not change appreciably with increasing c-Met concentrations and in the presence of highly concentrated serum proteins. This confirms that the specificity of the interaction between CLN3 and c-Met is also retained in a complex heterogeneous solution.

## Conclusions

Thanks to their exact replicability, solubility, and precise dye-conjugation, aptamers are detected by FCS with optimal single-molecule sensitivity, enabling a high-quality characterization. Our results highlight that FCS is ideal for the study of dye-conjugated aptamers targeting bulkier proteins, like c-Met or NCL, because the large change in molecular weight occurring upon complex formation results in a well-detected shift of the observed τ_D_. We estimated that the complex should have a molecular weight at least 3 times higher than free dye-conjugated aptamers to be quantitatively detected with this method, as for the 36t-PDGF-BB binding. This condition is fulfilled by a large variety of protein targets but precludes the observation of binding between aptamers and targets with lower molecular weight, which should be addressed differently, e.g., using a more complex dual-color fluorescence cross-correlation spectroscopy (FCCS) approach that requires labeling of the two species with different dyes.^43^

We showed that FCS easily measures *K*_D_ values in the low nM range, which is typical of many aptamer–protein pairs. However, sub-nM affinities can, in principle, be measured, provided that fluorophore brightness and photon detection efficiency are sufficiently high. We also showed that binding kinetics are effectively measured with FCS by monitoring binding equilibration. In this case, the sensitivity is ultimately limited by the acquisition time of a single autocorrelation curve, which restricts the applicability to slow equilibration processes (tens of minutes to hours). Faster kinetics (seconds to minutes) are unlikely to be well discriminated by the proposed method. Still, a remarkable advantage over many other analytical techniques is that FCS operates on homogeneous solutions and requires essentially a single step of sample handling, i.e., mixing aptamer and target. In this way, FCS circumvents technical issues associated with surface-immobilization of molecules, phase separation, gradients, washing steps, or the use of microfluidics. FCS thereby constitutes a useful validation and optimization tool, able to overcome some specific limitations of other methods. FCS is not well-suited for high-throughput measurements of affinity. Still, it can be readily applied to compare a restricted number of preselected aptamer sequences, to refine post-SELEX modifications, or for a more robust characterization.

Additionally, the detection of dye-conjugated CLN3 with FCS proved to be compatible with the presence of mM concentrations of serum proteins, enabling the investigation of aptamer behavior under conditions closer to the physiological situation. Our data demonstrated that CLN3 interaction with c-Met is unaffected by the presence of serum proteins. Additionally, CLN3 showed negligible interactions with albumin (*K*_D_ >300 μM) and other abundant serum proteins. Even if sensitivity is limited compared to pure solutions, mostly due to the increased viscosity, these results point out how FCS can support the study of aptamer behavior within complex biofluids.

## Supporting Information Available

Binding characterization of the aptamers AS1411 and 36t with FCS, diffusion of CLN3 exposed to IgG, Tf, and Fibr, results of control experiments, and additional equilibration kinetics data (PDF)
