# Supplementary material for: Fluorescence Correlation Spectroscopy as a Versatile Method to Define Aptamer–Protein Interactions with Single-Molecule Sensitivity
Source: Anal Chem. 2023 Dec 21;96(1):137–44. doi: 10.1021/acs.analchem.3c03341 (PMC10782416; doi:10.1021/acs.analchem.3c03341)
Supplement: Supplementary file 1 — ac3c03341_si_001.pdf [file ac3c03341_si_001.pdf]

Supporting Information

Fluorescence Correlation Spectroscopy as a Versatile Method to Define Aptamer-Protein Interactions with Single Molecule Sensitivity

David Porciani[1, 2, #], Manuela Maria Alampi[3], Stefania Abbruzzetti[3], Cristiano Viappiani[3], Pietro Delcanale[3, *]

[1]MU Bond Life Sciences Center, University of Missouri-Columbia, 1201 Rollins Street, Columbia MO 65211-7310 (USA)
[2]Department of Molecular Microbiology & Immunology, School of Medicine, University of Missouri-Columbia, 1 Hospital Dr, Columbia, MO 65212 (USA)
[3]Dipartimento di Scienze Matematiche, Fisiche e Informatiche, Università di Parma, Parco Area delle Scienze 7A, 43124, Parma, Italy

[#]present address: SomaLogic, Inc., La Jolla, CA 92037, USA

*Corresponding author:

Pietro Delcanale, e-mail: pietro.delcanale@unipr.it

ORCID

David Porciani: 0000-0001-7803-6546

Manuela Maria Alampi: 0000-0003-1996-4507

Stefania Abbruzzetti: 0000-0001-7685-8554

Cristiano Viappiani: 0000-0001-7470-4770

Pietro Delcanale: 0000-0001-8235-765X

Table of Contents

Figure S1	FCS autocorrelation curve of c-Met protein	S2
Figure S2	Brightness of CLN3 and CLN3 + c-Met	S3
Figure S3	Fluorescence anisotropy of CLN3 and CLN3 + c-Met	S4
Figure S4	FCS autocorrelation curves of AS1411 and 36t with their protein targets	S5
Figure S5	Fitting results of FCS autocorrelation curves for CLN3 + c-Met	S6
Figure S6	Binding affinity of AS1411-NCL and 36t-PDGF-BB complexes	S7
Figure S7	Additional equilibration kinetics for CLN3 + c-Met	S8
Figure S8	Control for stability of photon count rate during kinetic measurements	S9
Figure S9	Control measurements for background signal due to serum proteins	S10
Figure S10	Control measurement for effect of viscosity on observed τ_D	S11
Figure S11	Observed τ_D of CLN3 exposed to IgG, Tf and Fibr at high concentrations	S12
Figure S12	Control measurements for background signal due to Hb and serum	S13

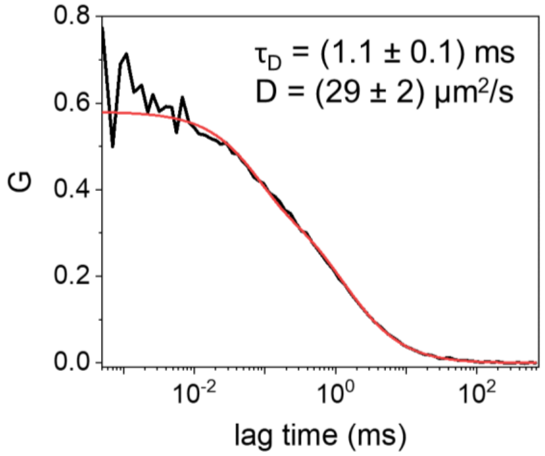

Figure S1. *FCS autocorrelation curve (black) obtained on a solution containing 100 nM of c-Met and ~1 nM of the small (~1 kDa) fluorescent affinity probe REDtrisNTA, that labels with high-affinity the polyhistidine tag of the recombinant c-Met. The large excess of c-Met ensures that the fluorescent probe is fully protein-bound. The red line shows the results of the fitting with a model comprising a single diffusing species, yielding the* τ_D *and D values of the c-Met protein. Reported values were obtained from 5 repetitions and are nicely consistent with those obtained for CLN3 exposed to an excess of c-Met.*

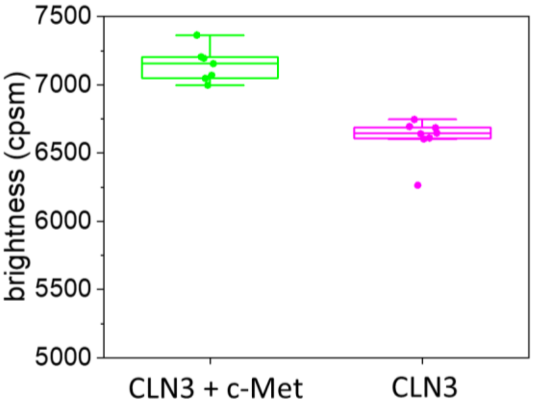

***Figure S2**. Comparison of measured brightness, expressed in photon counts per second per diffusing fluorescent species (cpsm) of CLN3-atto633 alone (magenta) or exposed to an excess of c-Met (green). The measured brightness values are very similar and consistent with a 1:1 binding stoichiometry. The small difference in observed brightness (~ 8%) is most likely related to uncertainties in the determination of the number of diffusing fluorescent species for the solution containing c-Met, or possibly to a small change in the fluorescence emission quantum yield of the fluorophore. Box represents the 25% to 75% percentile and whiskers the 10% to 90%.*

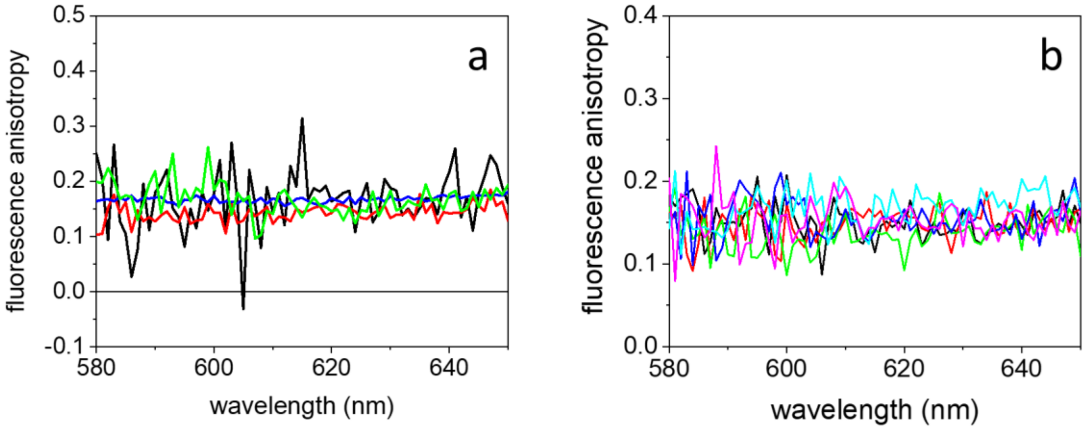

Figure S3. *(a) Fluorescence anisotropy spectra measured on solutions containing CLN3-atto633 at different concentrations: 100 nM (blue), 10 nM (red), 1 nM (black and green; the green spectrum was obtained with increased excitation and emission bandwidth compared to previous spectra). It can be noted that the signal becomes very noisy at concentrations of aptamers below 10 nM, preventing the measurements at aptamer concentrations lower than 1 nM. (b) Fluorescence anisotropy spectra measured on solutions containing 1 nM CLN3-atto633 and different concentrations of c-Met: 0 (black), 0.3 nM (red), 1 nM (green), 6 nM (blue), 25 nM (cyan), 100 nM (magenta). No appreciable changes in fluorescence anisotropy are observed in the presence of c-Met, even in large excess. Binding between CLN3 and c-Met is not detected by the present fluorescence anisotropy data. This is possibly due to a residual mobility of the fluorophore when complex is formed, and/or to the low signal-to-noise ratio which prevents the observation of anisotropy changes.*

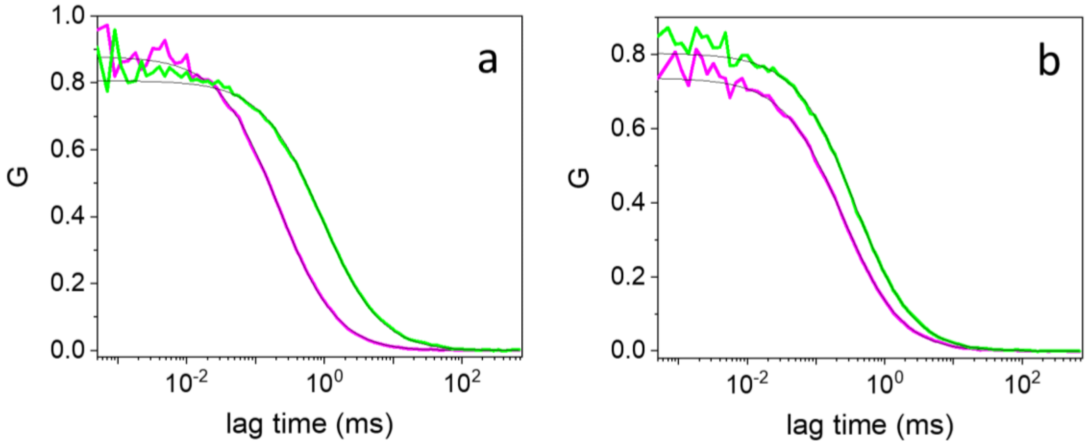

Figure S4. *(a) FCS autocorrelation curves measured for 1 nM AS1411-atto633 alone (magenta) and exposed to 500 nM of NLC (green). The black lines represent the results of the fitting with a model comprising a single diffusing species. A D ≈ 110 μm^2/s is measured for the free AS1411-atto633 aptamer, while the observed D decreases to ~30 μm^2/s in the presence of NCL, consistent with the formation of AS1411-NCL complexes.*
(b) FCS autocorrelation curves measured for 1 nM 36t-atto633 alone (magenta) and exposed to 200 nM of PDGF-BB (green). The black lines represent the results of the fitting with a model comprising a single diffusing species. A D ≈ 100 μm^2/s is measured for the free 36t-atto633 aptamer, while the observed D decreases to ~70 μm^2/s in the presence of PDGF-BB, consistent with the formation of 36t-PDGF-BB complexes.

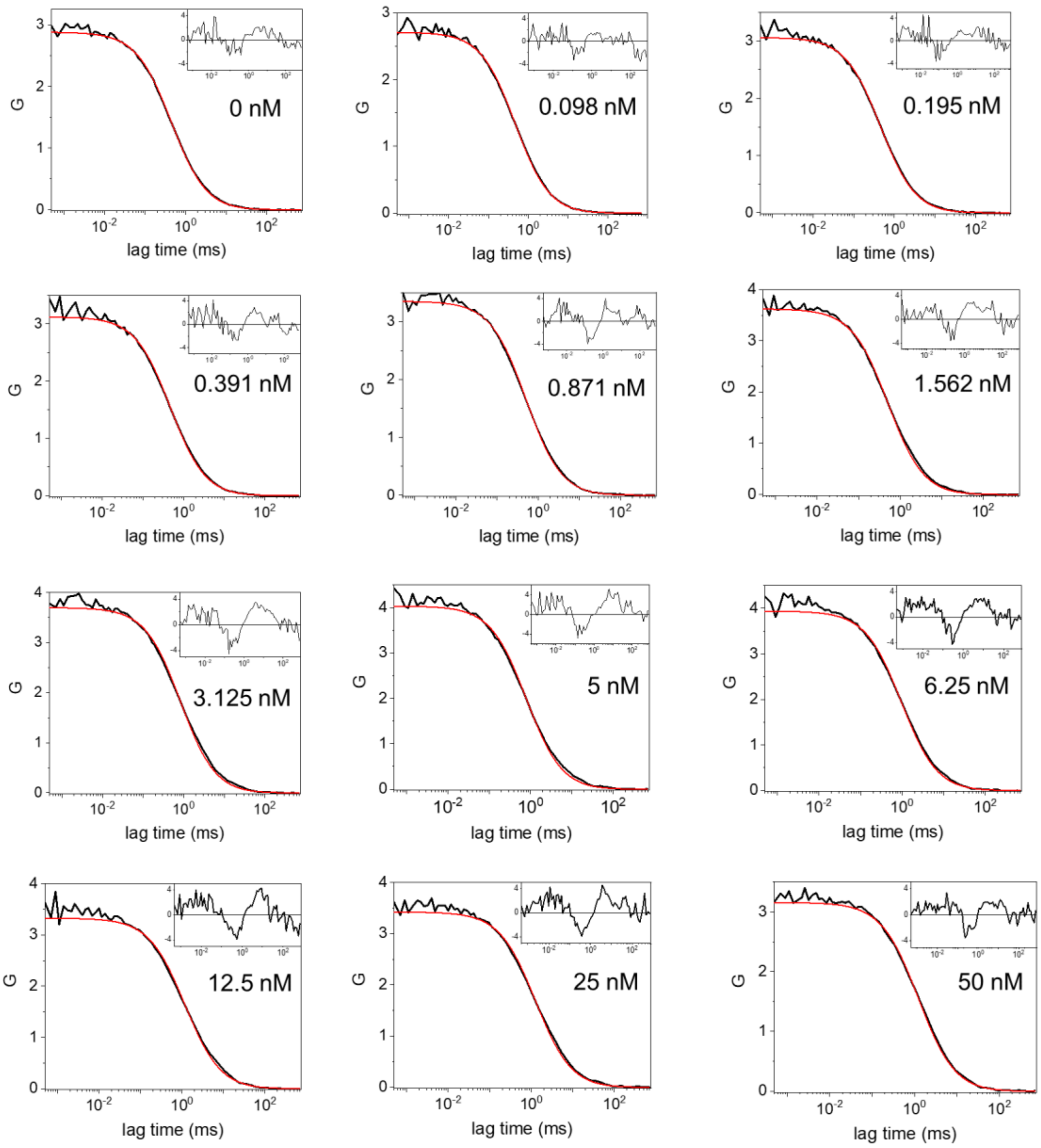

Figure S5. *FCS autocorrelation curves (black) and corresponding results obtained by the fitting with a model comprising a single effective diffusing (red) obtained for 100 pM of CLN3-atto633 and an increasing concentration of c-Met (indicated in the graph). The fitting residue curves are shown in the insets. These curves correspond to the data shown in Figure 2a.*

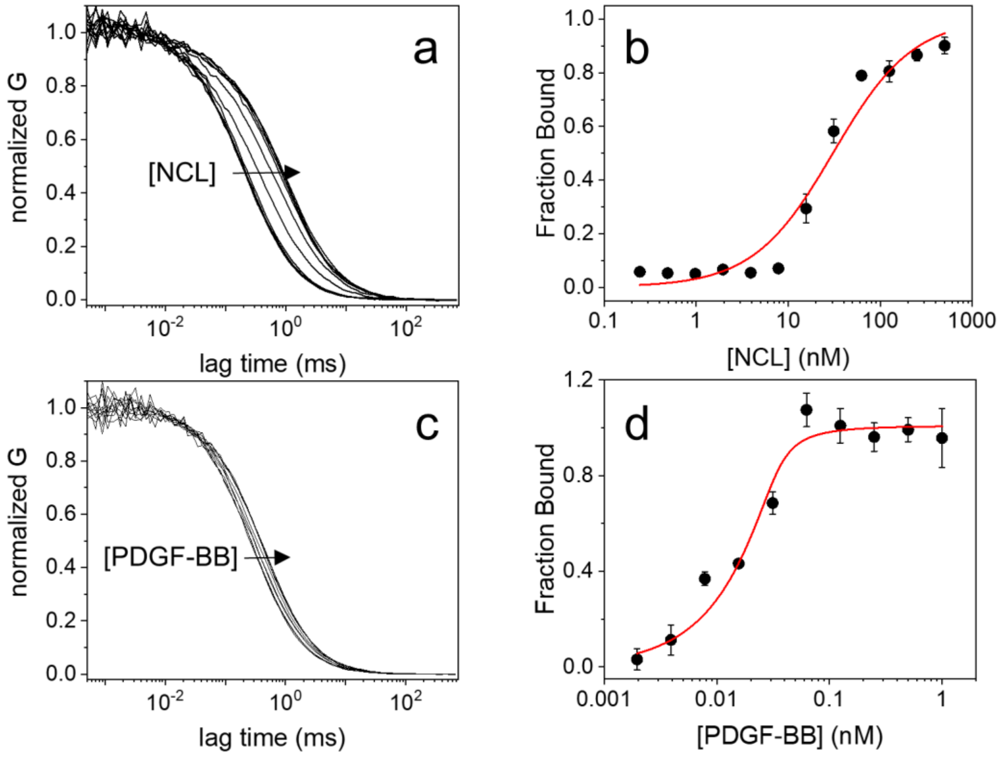

Figure S6. *(a) FCS autocorrelation curves, normalized to 1, measured on solutions containing 100 pM of AS1411-atto633 and an increasing concentration of NCL, from 0 to 500 nM. The change of the curves with NCL concentration is highlighted by the arrow. (b) Corresponding data showing the calculated fraction of AS1411-atto633 bound to NCL at increasing protein concentration (black). The red line shows the result of the fitting with a binding model, yielding a* K_D *= 30 nM.*

(c) FCS autocorrelation curves, normalized to 1, measured on solutions containing 100 pM of 36t-atto633 and an increasing concentration of PDGF-BB, from 0 to 1 nM. The change of the curves with PDGF-BB concentration is highlighted by the arrow. (d) Corresponding data showing the calculated fraction of 36t-atto633 bound to PDGF-BB at increasing protein concentration (black). The red line shows the result of the fitting with a binding model, yielding a K_D *in the pM range. A precise determination of* K_D *is not possible because experiment is likely performed under a titration regime ([36t-atto633] higher than real* K_D*). A more precise measurement would require a further dilution of the aptamer, below 100 pM. At such low concentrations the fluorescence intensity of 36t-atto633 approaches the background noise of the detectors.*

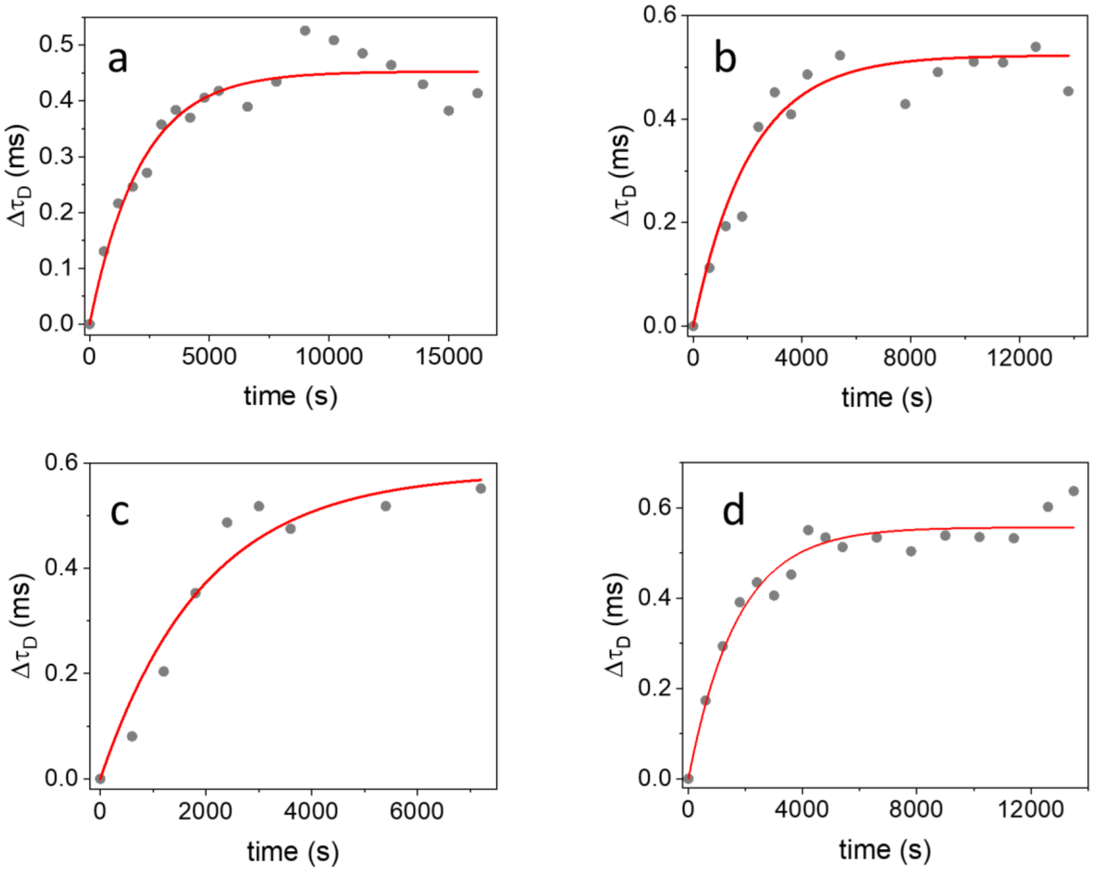

Figure S7. *Observed $\Delta\tau_D$ of the FCS autocorrelation curves measured at increasing time from the initial mixing of a solution containing 100 pM of CLN3-atto633 and 2 (a), 3 (b), 5 (c), 9 nM (d) of c-Met (black). The red line shows the result of the fitting with an exponential model, yielding the k_{equil} parameter.*

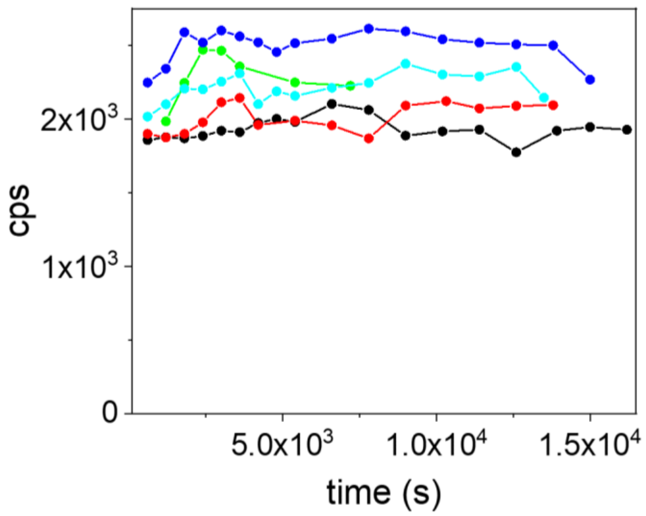

Figure S8. *Fluorescence photon counts per second (cps) detected in time-course measurements yielding kinetic parameters. Each point corresponds to an FCS acquisition carried out at increasing time from the initial mixing of the solutions. All data were obtained on solutions containing 100 pM of CLN3-atto633 and a constant concentration of c-Met: 2 nM (black), 3 nM (red), 5 nM (green), 7 nM (blue)and 9 nM (cyan).*
The data show that fluorescence remains stable during measurements, with minor fluctuations, confirming that kinetic measurements are not affected by dye photo-bleaching or non-specific surface interactions. The differences in absolute cps values between different solutions are due to experimental uncertainty in sample preparation or slightly different levels of dark cps of detectors.

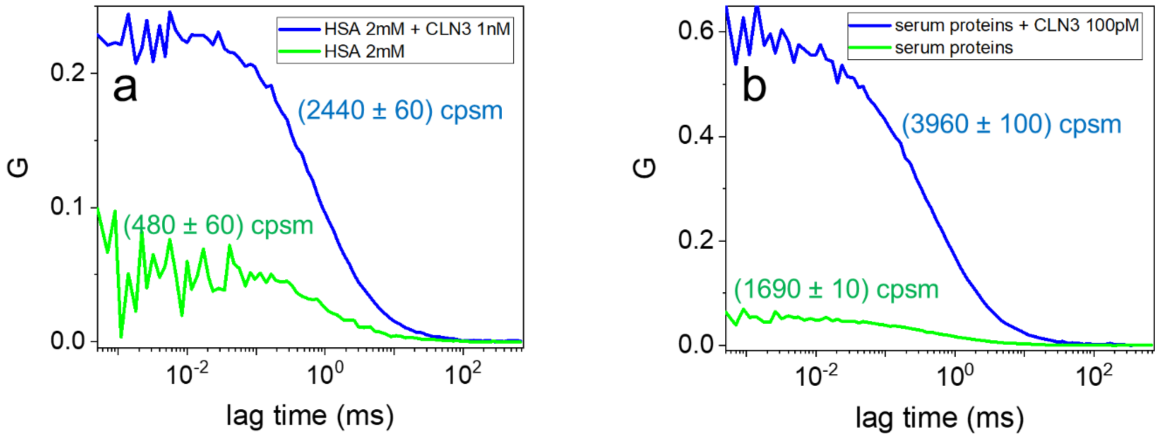

Figure S9. *(a) Comparison of FCS autocorrelation curves obtained on a solution containing 2 mM HSA alone (green) and with 1 nM CLN3-atto633 (blue). (b) Comparison of FCS autocorrelation curves obtained on a solution containing serum proteins alone (green) and with 100 pM CLN3-atto633 (blue). Corresponding brightness values, in photon counts per second per diffusing fluorescent species (cpsm), are reported next to the curves. These data show that, even if HSA or serum proteins are highly concentrated in solution, the FCS signal from CLN3-atto633 is well-detected above a background level arising from light scattering or weak protein auto-fluorescence.*

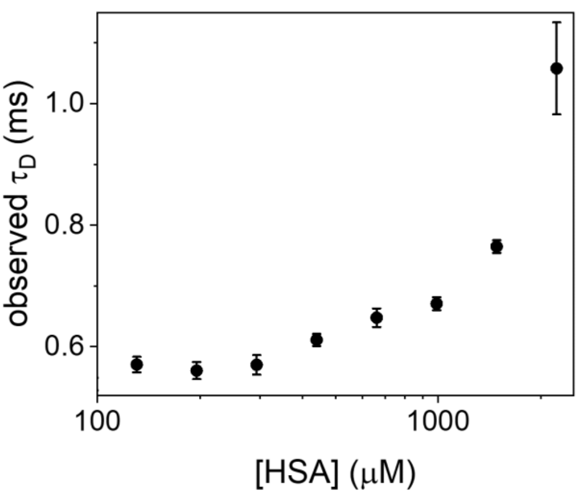

Figure S10. *Observed τ_D values obtained on solutions containing 1 nM of bovine serum albumin, conjugated with alexafluor647 dye, and an increasing concentration of HSA. The observed trend is qualitatively very similar to the one of CLN3 exposed to the same concentrations of HSA.*

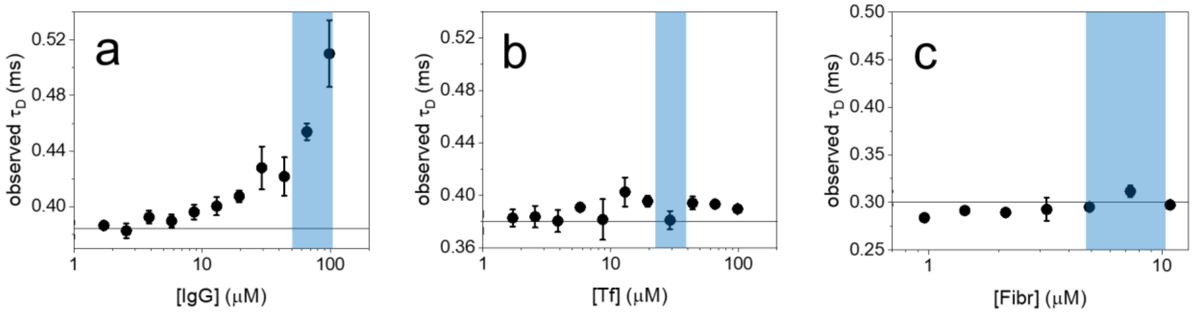

Figure S11. *Observed* τ_D *values obtained on solutions containing 1 nM of CLN3-atto633 and an increasing concentration of IgG (a), Tf (b) and Fibr (c). The highlighted regions indicate the range of protein concentration found in human serum.*

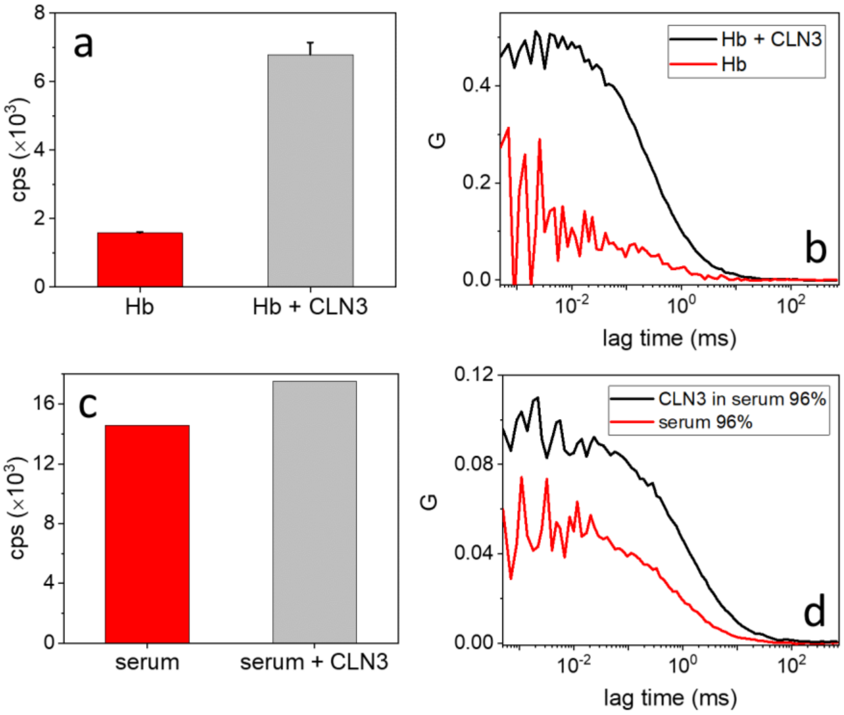

Figure S12. *(a) Comparison of photon counts per second (cps) detected by FCS for a solution containing 10 μM hemoglobin alone (red) and with 1 nM CLN3-atto633 (grey). (b) Comparison of FCS autocorrelation curves obtained on a solution containing 10 μM hemoglobin alone (red) and with 1 nM CLN3-atto633 (black). The data show that hemoglobin, that strongly absorbs visible light but is not fluorescent, has negligible effect on both cps and autocorrelation curves measured in the presence of aptamer.*
(c) Comparison of photon counts per second (cps) detected by FCS for a solution containing 96% fetal calf serum alone (red) and with 1 nM CLN3-atto633 (grey). (d) Comparison of FCS autocorrelation curves obtained on a solution containing 96% fetal calf serum alone (red) and with 1 nM CLN3-atto633 (black). Fetal calf serum 96% show a high cps value and a defined autocorrelation function, due to weakly fluorescent or scattering material. Still, a detectable change is observed when introducing 1 nM CLN3-atto633, suggesting that FCS potentially operates also in the presence of high concentrations of contaminants or in very concentrated biological fluids.
